# Author Correction: Thyroid hormone receptor beta (THRB) dependent regulation of diurnal hepatic lipid metabolism in adult male mice

**DOI:** 10.1038/s44324-024-00031-4

**Published:** 2024-09-02

**Authors:** Leonardo Vinicius Monteiro de Assis, Lisbeth Harder, Julica Inderhees, Olaf Jöhren, Jens Mittag, Henrik Oster

**Affiliations:** 1 Institute of Neurobiology, Center of Brain Behavior & Metabolism, University of Lübeck, Lübeck, Germany; 2 University Hospital Schleswig-Holstein, Campus Lübeck, Lübeck, Germany; 3 Bioanalytic Core Facility, Center of Brain Behavior & Metabolism, University of Lübeck, Lübeck, Germany; 4 Institute for Endocrinology and Diabetes–Molecular Endocrinology, Center of Brain Behavior & Metabolism, University of Lübeck, Lübeck, Germany; 5 Present Address: Division of Molecular Neurobiology, Department of Medical Biochemistry and Biophysics, Karolinska Institutet, Stockholm, Sweden

**Keywords:** Physiology, Metabolism, Systems biology, Endocrinology

Correction to: *npj Metabolic Health and Disease* 10.1038/s44324-024-00023-4, published online 13 August 2024

In this article, the funding information: “This work was supported by grants of the German Research Foundation (DFG) to HO 353-10/1, GRK-1957, and CRC/TR 296 “LOCOTACT” (ID 424957847, TP13 and TP14). The Metabolomics Workbench is supported by Metabolomics Workbench/National Metabolomics Data Repository (NMDR) (grant# U2C-DK119886), Common Fund Data Ecosystem (CFDE) (grant# 3OT2OD030544) and Metabolomics Consortium Coordinating Center (M3C) (grant# 1U2C-DK119889).” was omitted. The original article has been corrected.

