# Supplementary material for: Thyroid hormone receptor beta (THRB) dependent regulation of diurnal hepatic lipid metabolism in adult male mice
Source: NPJ Metab Health Dis. 2024 Aug 13;2:21. doi: 10.1038/s44324-024-00023-4 (PMC12118710; doi:10.1038/s44324-024-00023-4)
Supplement: Supplementary file 1 — Supplementary Information [file 44324_2024_23_MOESM1_ESM.pdf]

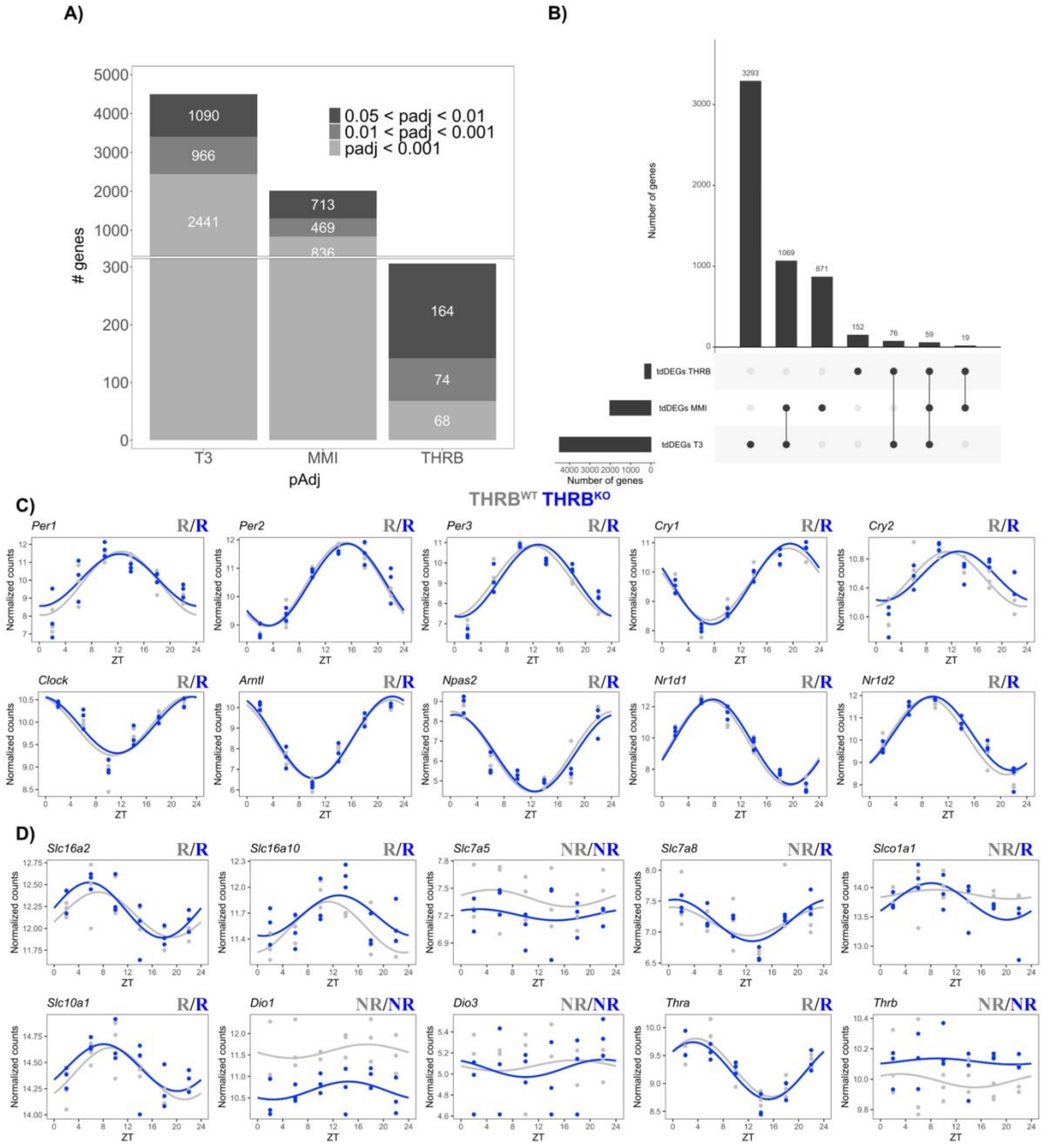

Supplementary Figure 1: Evaluation of core clock gene and thyroid hormone (TH) modulators in THRB^{WT} compared to THRB^{KO}. A) Comparison of time-dependent DEG numbers between livers from T_3- and MMI-treated and THRB-deficient animals stratified for significance threshold. B) UpSet plot of genes identified in panel A with a padj < 0.05). C) Core clock genes are shown. D) TH modulators, transporters, DIOs, and receptors are shown. Rhythm analysis performed by CircaCompare. N = 3 independent samples per ZT and condition.

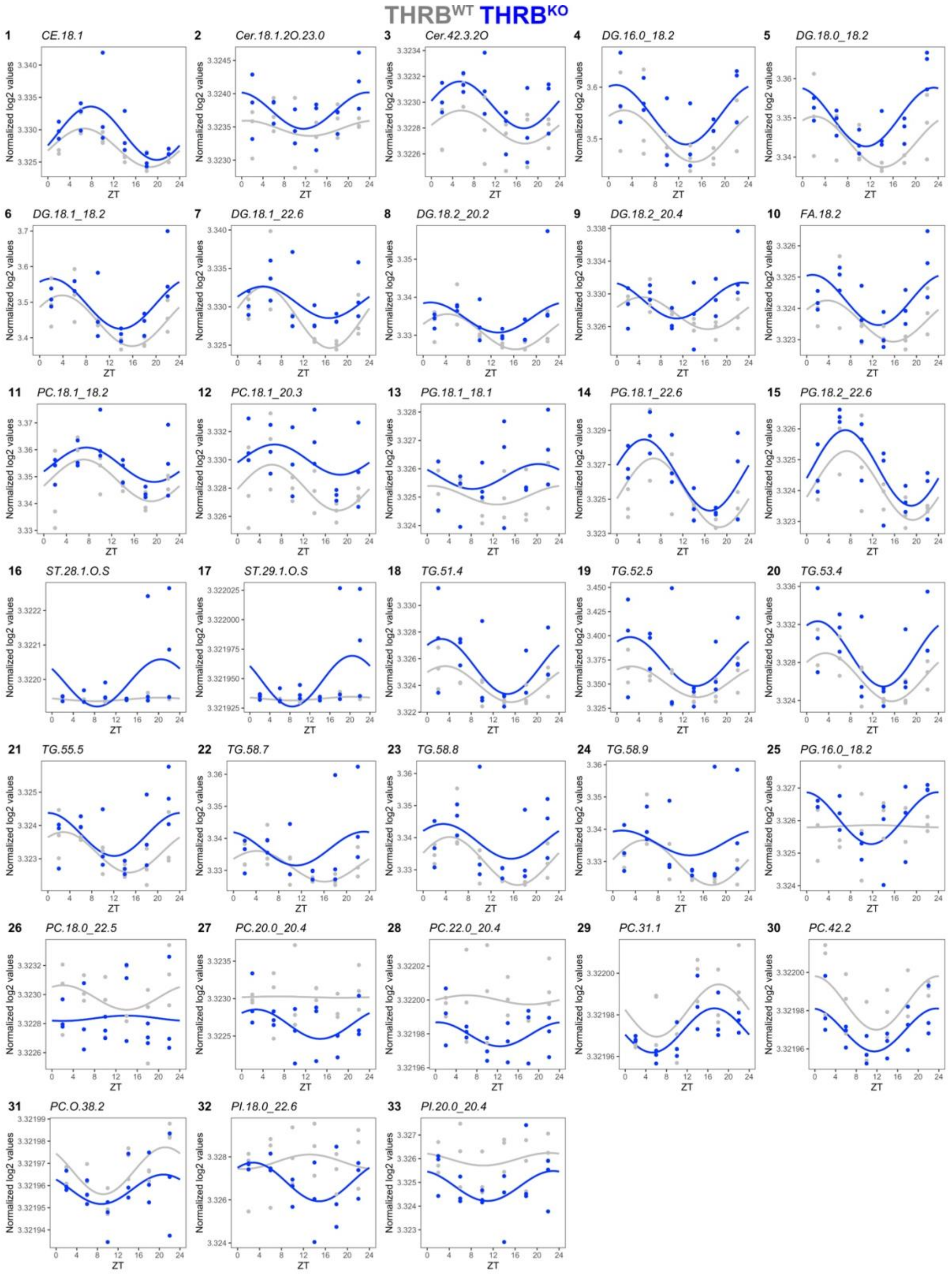

Supplementary Figure 2: Rewiring of liver lipidome in the absence of THRB. All lipids that show rhythm parameter alteration are depicted. Rhythm analysis performed by CircaCompare. N = 3 independent samples per ZT and condition.

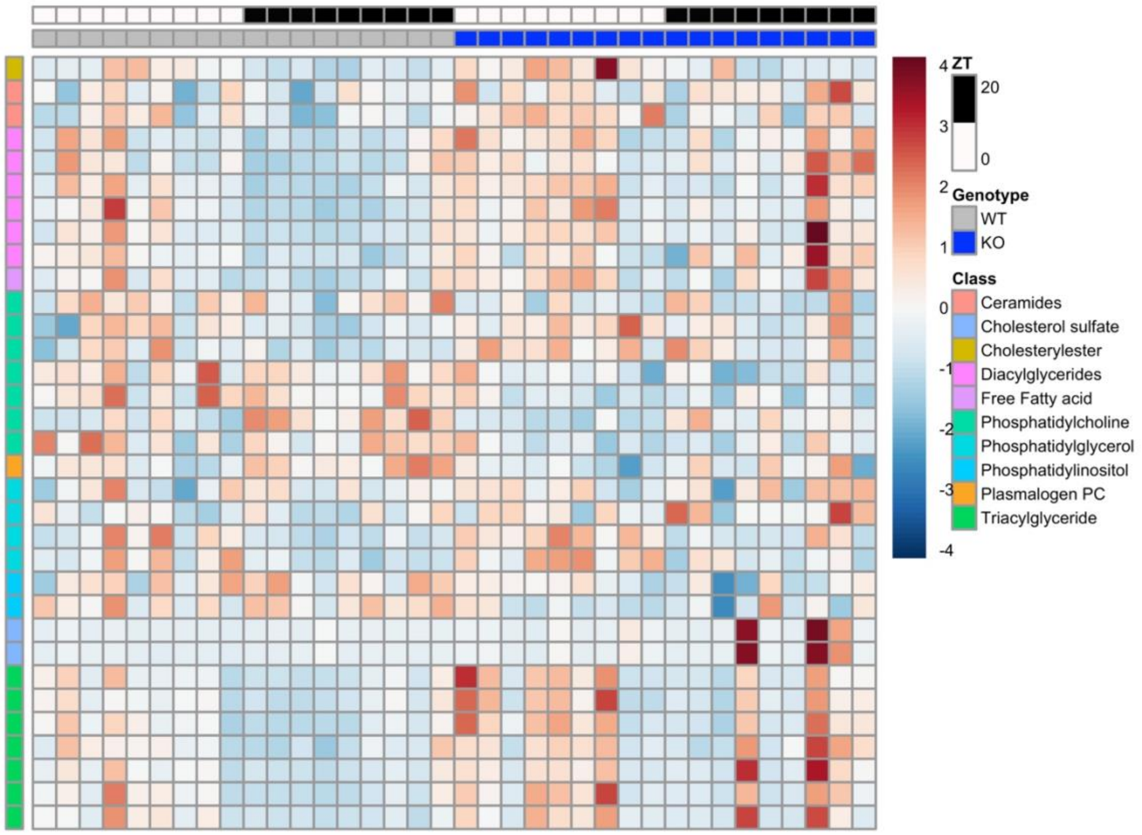

Supplementary Figure 3: Visualization of the rewiring of liver lipidome in the absence of THRB. Heatmaps show all lipids that show rhythm parameter alteration. Rhythm analysis performed by CircaCompare. N = 3 independent samples per ZT and condition.

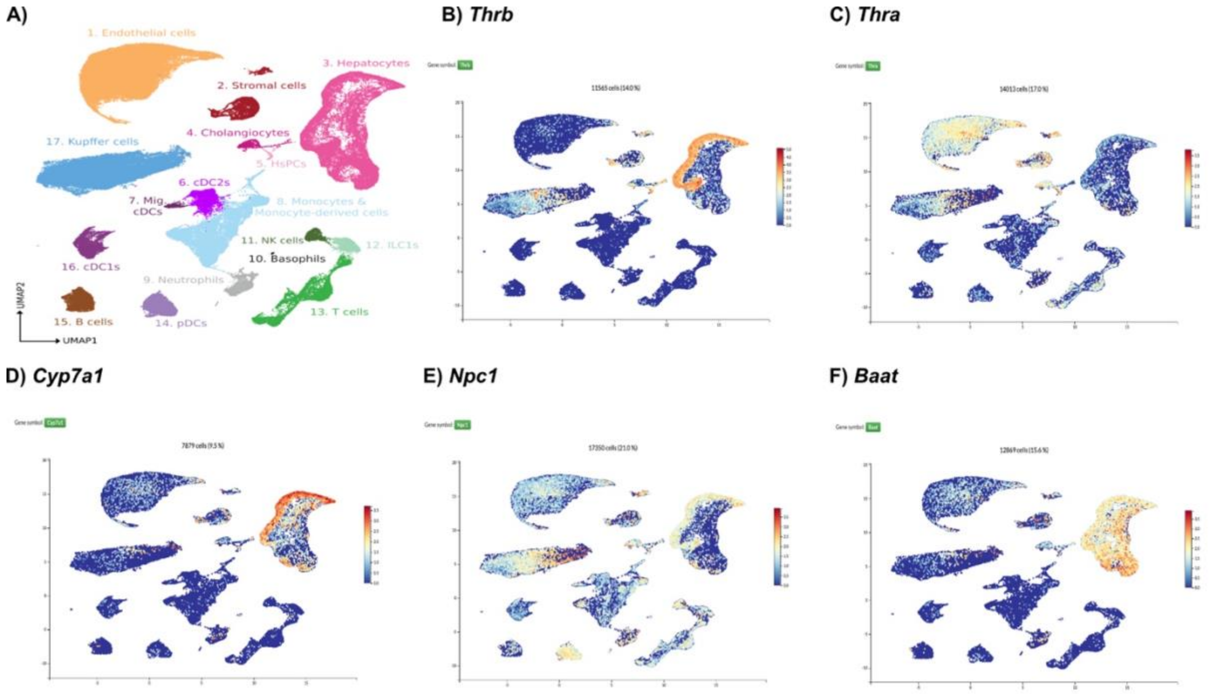

Supplementary Figure 4: Exploration of THRA and THRB signal in liver cells using public single-cell RNAseq database. A – F) Liver Cell Atlas (https://www.livercellatlas.org/index.php) was accessed and parameters were the following: no cut-off and order dots were used.

Supplementary data 1: Differentially expressed genes (DEGs) analysis.

Supplementary data 2: Transcriptome rhythm analysis

Supplementary data 3: Differentially rhythmic genes (DRGs) analysis

Supplementary data 4: Differentially lipidome rhythm analysis
